# Methodological Challenges in Predicting Periprosthetic Joint Infection Treatment Outcomes: A Narrative Review

**DOI:** 10.3389/fresc.2022.824281

**Published:** 2022-07-11

**Authors:** Elise Naufal, Marjan Wouthuyzen-Bakker, Sina Babazadeh, Jarrad Stevens, Peter F. M. Choong, Michelle M. Dowsey

**Affiliations:** ^1^Department of Surgery, St Vincent's Hospital Melbourne, University of Melbourne, Fitzroy, VIC, Australia; ^2^Department of Medical Microbiology and Infection Prevention, University Medical Center Groningen, University of Groningen, Groningen, Netherlands; ^3^Department of Orthopaedics, St Vincent's Hospital Melbourne, Fitzroy, VIC, Australia

**Keywords:** periprosthetic joint infection (PJI), arthroplasty, risk prediction, prognostic models, predictive models

## Abstract

The management of periprosthetic joint infection (PJI) generally requires both surgical intervention and targeted antimicrobial therapy. Decisions regarding surgical management–whether it be irrigation and debridement, one-stage revision, or two-stage revision–must take into consideration an array of factors. These include the timing and duration of symptoms, clinical characteristics of the patient, and antimicrobial susceptibilities of the microorganism(s) involved. Moreover, decisions relating to surgical management must consider clinical factors associated with the health of the patient, alongside the patient's preferences. These decisions are further complicated by concerns beyond mere eradication of the infection, such as the level of improvement in quality of life related to management strategies. To better understand the probability of successful surgical treatment of a PJI, several predictive tools have been developed over the past decade. This narrative review provides an overview of available clinical prediction models that aim to guide treatment decisions for patients with periprosthetic joint infection, and highlights key challenges to reliably implementing these tools in clinical practice.

## Introduction

Periprosthetic joint infections (PJI) are catastrophic complications of hip or knee arthroplasty with substantial long-term sequelae (1, 2). The rate of PJI has remained stable over recent decades, ranging from one to two percent after primary hip or knee arthroplasty (3, 4). However, the number of patients requiring treatment for PJI will increase in coming years as the demand for Total Joint Arthroplasty (TJA) steadily increases (3). When compared to aseptic revision, revision due to PJI is independently associated with a five-fold greater risk of mortality within 5 years of the procedure, with most of this excess mortality occurring within the first year (5). The diagnosis and treatment of PJI is resource-intensive, (6) and in the United States alone the annual direct cost of PJI management is expected to exceed $1.85 billion by the end of this decade (7).

Management of PJI generally requires surgical intervention alongside targeted antimicrobial therapy (8). Selecting the appropriate strategy for surgical management of a given infection–whether it be irrigation and debridement (I and D), one-stage or two-stage revision–must weigh the likelihood of successfully eradicating the infection against the potential treatment burden on the patient. It is worth noting that none of the most widely cited predictive models examine outcomes of arthrodesis or excision arthroplasty, as these are usually reserved for septic treatment failure or when the patient's condition precludes staged revision or I and D (9). Decisions relating to surgical management are complex, as they must take into consideration factors such as the timing and duration of symptoms, and the type and antimicrobial susceptibilities of the microorganism(s). Moreover, clinical factors, patient's preferences, and expectations of improvement in quality of life should also be considered.

Over the past decade, a number of tools that aim to predict the likelihood of success or failure of a given treatment strategy have been created to help clinicians and patients make informed decisions about the preferred strategy for surgical management of a given infection (10–17). However, the process of developing a clinical tool that can be reliably implemented remains challenging. This narrative review aims to provide a critical overview of these challenges, by focusing on the pathway from model development through to implementation. We present a summary of commonly used PJI prediction tools, detail some of the key issues that these tools face, and discuss the difficulties associated with collecting high-quality data on sufficiently large samples of patients experiencing this rare but devastating complication. We conclude by discussing the central role that standardized data collection through collaborative research groups can play in overcoming many of these challenges.

## Available Tools to Predict Treatment Outcomes of Periprosthetic Joint Infection

To understand the methodological challenges faced by researchers that are developing tools that predict PJI outcomes, we identified the five most widely cited of these tools (see Appendix A for search strategy) and detailed their development and validation process (Table 1). Of these tools, one predicts outcomes for patients treated with two-stage revision, (12) one predicts outcomes in patients undergoing either revision procedures or I&D (13) and three predict outcomes following I and D (10, 11, 15). All of these tools were published over the last decade, with three developed in American cohorts and two in European. The development and performance of these models is discussed in detail below. It is worth noting that there is an emerging capacity for machine learning to be used in the development of predictive tools (22). However, as none of the most widely cited tools have used such methods–and because the unique challenges of implementing machine learning techniques warrant careful individual attention–we have limited our discussion to models that rely upon traditional statistical techniques, such as logistic and cox regression.

**Table 1:** Five most widely cited tools to predict treatment outcomes of periprosthetic joint infection.

				**Target population**		**Model performance summary**	
**Authors**	**Sample size (events)**	No. of candidate predictor^*^	No. of predictors^**^	**Surgical treatment**	**Infection type**	**Internal validation**	**External validation**
Buller et al. (11)	309 (149)	30	18	I and D with polyethylene exchange	All PJI	C-Index 0.645	-
Tornero et al. (10)	222 (52)	48	5	I and D	Early PJI	AUC - 0.84 (95% CI 0.77–0.91)	AUC−0.64 (95%CI 0.59–0.69) (18) AUC 0.76 (95%CI 0.57–0.96) (19) AUC 0.72 (*p* = 0.01) (16) AUC 0.54 (95%CI 0.43–0.65) (20)
Sabry et al. (12)	314 (105)	39	15	Two-stage revision	All PJI	C-Index 0.773	-
Wouthuyzen-Bakker et al. (15)	340 (153)	34	7	I and D	Late-acute PJI	-	AUC 0.61 (95%CI 0.50–0.73) (20)
Kheir et al. (13)	1,438 (543)	75	11	I and D or revision	All PJI	AUC−0.69 (95% CI 0.65–0.73)	AUC 0.80 (95%CI 0.70–0.90) (21) AUC 0.60 (95%CI 0.50–0.69) (21)

*I and D, Irrigation and Debridement; AUC, Area under receiver operating characteristic curve; C-Index, Concordance Index*.

* *These represent the estimated number of candidate prediction parameters for each model. The number of prediction parameters may exceed the number of variables assessed for inclusion when categorical variables had more than two levels. The results presented here should be taken as estimates due to inconsistent reporting on how candidate predictors were parameterised in the included studies*.

** *This represents the number of prediction parameters in the final predictive model. When a simplified model was presented as the final model, this was used to determine the final number of prediction parameters*.

## The Process of Prediction: Model Development and Validation

To produce reliable estimates of the risk of an outcome event, such as infection eradication, a multivariable prediction model must be carefully developed and rigorously (internally and externally) validated. Developing a model begins with selecting an outcome of interest, and the identification of relevant candidate predictors (23). These candidate predictors are usually drawn from a patient's demographics, clinical history, clinical presentation and the characteristics of the disease (24). Identification of such variables is generally informed by consultation with clinical experts and prior work examining factors associated with the outcome of interest (25). Once the candidate predictors have been selected, they can then be included in a multivariable model–such as a logistic regression mode–owever, the process of how variables should be selected in the final model remains controversial (26).

The final multivariable model produces regression estimates (e.g., log odd ratios) for each included predictor along with an intercept value. The regression estimates indicate the independent contribution of each predictor to the estimated risk of the outcome, while the intercept provides an estimate of the baseline risk for an individual with all predictor values being equal to zero. The reliability of the model's predictive ability must then be examined by assessing how well the model distinguishes between those who do and others who do not experience the outcome of interest (i.e., model discrimination), and comparing the predicted and observed outcome frequencies within patient subgroups (i.e., model calibration) (24, 27, 28).

While the reliability of a model's predictive ability is often assessed using the exact dataset from which it was developed, this approach will generally overestimate how well the model will perform in samples of new patients (24, 27). This problem arises from the fact that models are specifically designed to optimally fit their development dataset (28). Importantly, concerns about the possibility of such “overfitting” are most prominent when there are few outcomes in the development data, as is often the case when examining the treatment of a rare complication such as PJI. The first step to address this concern is to internally validate the predictive model's performance in a subset of the cohort which the model was developed, which is done by randomly splitting the sample into a development and validation datasets (28). The model is then used to estimate predicted outcomes for patients in the validation dataset. This allows for comparisons to be made between the predicted and actual outcomes in a sample of patients that is distinct from the training sample. Where available data are limited, re-sampling methods such as bootstrapping or cross-validation may be used to assess performance on random sub-samples of the training dataset (28). To ensure that the model has clinical value in other settings, it needs to be externally validated in independent cohorts (29, 30). This is because even if the model is methodologically robust, the new sample or clinical setting may be too different for it to be applicable for that practice (31). For instance, different settings may exhibit distinct patterns of antimicrobial resistance or comorbidity. Despite the central role that they play in understanding whether a published model is of value in practice, high-quality external validations studies remain uncommon (32).

Over the past decade, several predictive models have been developed to assist patients and clinicians to better understand the likely outcome of PJI treatment (10–17). However, performance has varied, even among the most widely cited of these models (see Table 1). In development or internal validation datasets, only two models have reported a concordance index (C-index) or area under receiver operating characteristic curve (AUC) above the standard threshold for acceptable discrimination, which is generally set at 0.70 (10, 12). Of the two models that initially reported acceptable discriminative ability, only the KLIC score (which stands for Kidney, Liver, Index surgery, C-reactive protein) has been externally validated. The KLIC score which aims to predict outcomes for patients with early PJI treated with I and D has been validated in four external cohorts, though the performance of the model has varied substantially from having almost no discriminative ability (AUC 0.54, 95%CI 0.43–0.65) to having acceptable performance (AUC 0.76, 95%CI 0.57–0.96) (18–20, 33). Across these validation studies, the KLIC score was shown to be most useful when estimating risk in patients with very low or very high scores, though it had limited predictive value for other patients (18, 33). Two other predictive tools have been externally validated. The CRIME-80 score (which stands for COPD, CRP >150 mg/L, Rheumatoid Arthritis, Indication prosthesis: fracture, Male, Exchange of mobile components and Age 80 years) was shown to have relatively poor discriminative performance (AUC 0.61, 95% CI 0.50–0.73) (20). The model developed by Kheir et al. showed a more varied performance when externally validated on two datasets (AUC 0.80 95% CI 0.70–0.90 and AUC 0.60 95% CI 0.50–0.69) (21). Importantly, to date no published external validations of these predictive tools have reported on the calibration of these models.

## Challenges of Developing and Validating Models to Predict PJI Outcomes

### Development and Validation in Sufficiently Large Samples of Patients

When creating a predictive model, it is vital that the development dataset includes a sufficient number of patients overall and a sufficient number of patients experiencing the outcome of interest (23, 26, 28). While the appropriate way to determine the necessary sample for developing a predictive model remains a matter of debate, the most commonly cited approach is to ensure that at least 10 patients in the cohort have experienced the outcome of interest for each predictor that is considered for inclusion in the final model (26). None of the most widely cited PJI prediction models have satisfied this criterion, (10–13, 15) which is commonly called the “events per prediction parameter” (EPP) rule, when it is appropriately applied to the number of predictors that are considered for inclusion in the model (26, 34). Even if available tools had adhered to this rule of thumb, in many instances this approach will underestimate the sample size needed for an accurate and reliable model (26). Some have suggested increasing the stringency of this requirement to up to 50 events per predictor, (35) while others have highlighted that the true sample size required to develop a model for a specific clinical context must consider the number of events per prediction parameter alongside the total sample size, the incidence of the outcome event, and the desired performance of the model (26). No matter which of these methods is employed, it is evident that the availability of sufficiently large samples remains a challenge for those hoping to develop robust models to predict PJI treatment outcomes.

While the availability of sufficiently large samples has–and continues to–pose a challenge for those aiming to develop prediction tools for PJI treatment outcomes, it also presents a significant challenge for those attempting to validate available tools. To date, there has been little research published on the size of samples needed to perform a robust external validation of a predictive tool (36). The results of available simulation studies have suggested that external validation datasets should contain 100 events as a bare minimum, though reliable estimates of model performance may require analysis of validation datasets with a minimum of between 200 and 500 events (32, 36). As the sample size required for these validations is dependent on the treatment failure rate of the cohort, it is difficult to estimate the overall number of patients required for an accurate validation. To date, only one available external validation study of a PJI treatment outcome prediction tool has examined a sample in which more than 100 outcome events were recorded (18). This study by Lowik et al. included 148 outcome events, while other available external validation studies have examined samples with between nine and fifty-two outcome events (16, 18, 20, 33). Given that external validations of prediction tools discussed in this paper have generally relied upon small sample sizes, it can be expected that most available validation studies are susceptible to providing exaggerated or misleading estimates that may overestimate the actual predictive performance of the tool (37).

### Practical Constraints When Collecting Data on Relevant Predictors

The outcome of a given approach in managing a PJI is influenced by a complex array of factors relating to the patient's medical history, their index arthroplasty procedure, previous attempts to treat the infection, the clinical and microbiological features of the infection, and the characteristics of the planned procedure (see Table 2). Collecting data on all potentially relevant predictors poses a substantial practical challenge, as it generally requires time intensive prospective data collection or extensive chart reviews. This practical constraint likely contributes to the challenge of collecting data on large samples of patients that are being treated for this rare complication. However, it also poses important challenges for the quality of data that has been used in some published validation studies, with many of these studies reporting substantial amounts of missing variables that resulted from reliance on retrospective collection of data from existing medical records (20, 33). While methods such as multiple imputation can address issues relating to missing data in development and validation research, (38, 39) the usability of these models in practice is dependent upon clinicians having access to the complete parameters of the predictive model when key treatment decisions are being made. As an example of this, many of the diagnostic tests used to isolate the causative microorganisms are often collected intraoperatively during the revision or I and D procedure. This limits the practical value of these tools, which all aim to help guide decisions about optimal surgical management, as it does not allow for clinicians to fully complete all parameters of the model prior to decisions about surgical management being made (22).

**Table 2:** Predictors included in the five most widely cited tools to predict treatment outcomes of periprosthetic joint infection.

	**Buller**	**Tornero**	**Sabry**	**Wouthuyzen-Bakker**	**Kheir**
**Basic clinical information**				
Age					
Sex					
Smoking status					
Body mass index					
ASA physical status classification					
**Characteristics of index arthroplasty**				
Joint replaced (hip vs. knee)					
Indication for index surgery					
Use of cemented prosthesis					
Previous revision(s) or infection(s)					
Number previous surgeries					
**Comorbidities**				
Diabetes					
Immunocompromised					
Heart disease					
Chronic renal failure					
Cirrhosis of the liver					
Rheumatoid arthritis					
History of myocardial infarction					
**Clinical presentation of infection**				
Time from index surgery					
Duration of symptoms					
Presence of sinus tract					
**Pathology and microbiology**				
Type of infecting organism					
Presence of resistant organism					
ESR					
WBC					
Hemoglobin					
C-reactive protein levels					
**Characteristics of treatment strategy**				
Surgery type (I and D, 1-stage, 2- Stage)					
Exchanging mobile components					
Soft tissue coverage required					

*ASA, American Society of Anaesthesiologists; ESR, Erythrocyte Sedimentation Rate; WBC, White Blood Cells*.

### Beyond Model Performance: Measuring the True Value of Predictive Tools

Overcoming the challenges outlined above is likely to result in more robust and accurate predictive models being available for use in clinical practice. However, the true value of predictive tools is not determined by metrics of model performance, but by whether they can improve patient outcomes when implemented in practice (31, 40). While some might assume that providing accurate estimates of risk should improve clinical decision making, the relationship between model performance and improving patient outcomes can be influenced by a number of factors. The practical impact of such a tool may be influenced by its usability or by low levels of uptake by clinicians. It may also be influenced by new information inadvertently distorting the patients' or clinicians' decision-making process (e.g., through contributing to anchoring or framing bias) (41). Despite this, no published studies have attempted to examine the clinical or economic impact of implementing any available tools to predict PJI treatment outcomes in clinical practice.

While estimates of the potential value of a predictive tool may be assessed through processes such as decision modeling, (40) as with any healthcare intervention the effect of implementing these tools in clinical practice should ideally be assessed in (cluster) randomized trials (30). In the absence of such trials, it is not possible to establish whether the use of tools to predict PJI treatment outcomes are likely to improve (or possibly harm) patient outcomes or allow for more efficient allocation of healthcare resources. This is especially important as no available PJI treatment prediction tools have reported levels of discrimination and calibration that are sufficiently high to justify the use of these tools without rigorous clinical evaluation. However, as is the case with most research aiming to improve PJI outcomes, the rarity of this complication means that such evaluations are likely to require substantial time and resources, along with collaboration across several centers.

## Conclusion

The promise of truly individualized treatment that improves patients' outcomes and reduces the burden associated with treating PJI, provides a strong rationale for the ongoing efforts to develop reliable predictive tools that can be implemented in clinical practice. However, if this goal is to be achieved, the important practical and methodological challenges outlined in this review must first be overcome. Many of these challenges can be traced directly back to the relative rarity of this devastating complication. Because of this, these challenges are unlikely to be overcome by researchers and clinicians working in isolation. Ongoing collaboration between research groups offers an opportunity to not only increase the sample available for the development and validation of robust models, it also allows for the standardization of definitions and data collection instruments across centers. Moreover, it encourages pooling of clinical and statistical expertise to ensure that future models are as impactful as possible when implemented in everyday practice (42). It is with these considerations in mind – and with the broader aim of improving outcomes for patients that experience periprosthetic joint infection – that the Orthopedic Device Infection Network (ODIN) (www.odininternational.org) has been established as a means of facilitating international collaboration with sites across Australia, Europe, the United States and New Zealand.

## Author Contributions

EN wrote the first draft. All authors contributed to the conception of the review, revising the manuscript for critically important intellectual content, read, and approved the submitted version.

## Conflict of Interest

The authors declare that the research was conducted in the absence of any commercial or financial relationships that could be construed as a potential conflict of interest.

## Publisher's Note

All claims expressed in this article are solely those of the authors and do not necessarily represent those of their affiliated organizations, or those of the publisher, the editors and the reviewers. Any product that may be evaluated in this article, or claim that may be made by its manufacturer, is not guaranteed or endorsed by the publisher.
