# Supplementary material for: Methodological Challenges in Predicting Periprosthetic Joint Infection Treatment Outcomes: A Narrative Review
Source: Front Rehabil Sci. 2022 Jul 11;3:824281. doi: 10.3389/fresc.2022.824281 (PMC9397789; doi:10.3389/fresc.2022.824281)
Supplement: Supplementary file 1 [file Table_3.DOCX]

**Appendix A**

We aimed to identify the five most widely cited predictive tools that examine outcomes of surgical management of PJI. Models developed using ensemble/machine learning techniques were excluded. These tools were identified by searching Scopus on 08/06/2022 using the search terms below. The retrieved entries (n=860) were sorted according to citation count, and titles and abstracts were screened until five eligible studies were identified. As all external validation studies are expected to cite the development paper, external validation studies were identified by searching the “cited by” articles for each of the included models.

**Search term**

( TITLE-ABS-KEY ( "prosthetic joint infection*" OR "periprosthetic joint infection* " OR "prosthetic infection*" OR "periprosthetic infection*" OR pji ) ) AND ( ( TITLE-ABS-KEY ( ( multivaria* OR predict* OR prognos* OR risk ) W/3 ( analysis OR tool* OR calculat* OR score* OR model* OR algorithm* ) ) ) OR ( TITLE-ABS-KEY ( ( "c statistic" OR c-statistic OR discrimination OR calibration OR auc OR "area under the curve" OR "area under the receiver operator characteristic curve" OR auroc ) ) ) OR ( TITLE-ABS-KEY ( ( ( internal* OR external* OR model* ) W/3 ( validat* OR develop* ) ) ) )

| **Citation count** | |
| --- | --- |
| **Reference** | **Number of citations** |
| Buller LT, Sabry FY, Easton RW, Klika AK, Barsoum WK. The preoperative prediction of success following irrigation and debridement with polyethylene exchange for hip and knee prosthetic joint infections. Journal of Arthroplasty. 2012;27(6):857-64 e1-4. | 109 |
| Sabry FY, Buller L, Ahmed S, Klika AK, Barsoum WK. Preoperative prediction of failure following two-stage revision for knee prosthetic joint infections. Journal of Arthroplasty. 2014;29(1):115-21. | 49 |
| Tornero E, Morata L, Martinez-Pastor JC, Bori G, Climent C, Garcia-Velez DM, et al. KLIC-score for predicting early failure in prosthetic joint infections treated with debridement, implant retention and antibiotics. Clin Microbiol Infect. 2015;21(8):786 e9- e17. | 43 |
| Wouthuyzen-Bakker M, Sebillotte M, Lomas J, Taylor A, Palomares EB, Murillo O, et al. Clinical outcome and risk factors for failure in late acute prosthetic joint infections treated with debridement and implant retention. J Infect. 2019;78(1):40-7. | 40 |
| Kheir MM, Tan TL, George J, Higuera CA, Maltenfort MG, Parvizi J. Development and Evaluation of a Prognostic Calculator for the Surgical Treatment of Periprosthetic Joint Infection. J Arthroplasty. 2018;33(9):2986-92 e1. | 21 |
